# Quantifying the Elastic Property of Nine Thigh Muscles Using Magnetic Resonance Elastography

**DOI:** 10.1371/journal.pone.0138873

**Published:** 2015-09-23

**Authors:** Mashhour K. Chakouch, Fabrice Charleux, Sabine F. Bensamoun

**Affiliations:** 1 Biomechanics and Bioengineering Laboratory, UMR CNRS 7338, Sorbonne University, Université de Technologie de Compiègne, Compiègne, France; 2 ACRIM-Polyclinique Saint Côme, Compiègne, France; Faculty of Animal Sciences and Food Engineering, University of São Paulo, BRAZIL

## Abstract

**Background:**

Pathologies of the muscles can manifest different physiological and functional changes. To adapt treatment, it is necessary to characterize the elastic property (shear modulus) of single muscles. Previous studies have used magnetic resonance elastography (MRE), a technique based on MRI technology, to analyze the mechanical behavior of healthy and pathological muscles. The purpose of this study was to develop protocols using MRE to determine the shear modulus of nine thigh muscles at rest.

**Methods:**

Twenty-nine healthy volunteers (mean age = 26 ± 3.41 years) with no muscle abnormalities underwent MRE tests (1.5 T MRI). Five MRE protocols were developed to quantify the shear moduli of the nine following thigh muscles at rest: rectus femoris (RF), vastus medialis (VM), vastus intermedius (VI), vastus lateralis (VL), sartorius (Sr), gracilis (Gr), semimembranosus (SM), semitendinosus (ST), and biceps (BC). In addition, the shear modulus of the subcutaneous adipose tissue was analyzed.

**Results:**

The gracilis, sartorius, and semitendinosus muscles revealed a significantly higher shear modulus (μ__Gr_ = 6.15 ± 0.45 kPa, μ__ Sr_ = 5.15 ± 0.19 kPa, and μ__ ST_ = 5.32 ± 0.10 kPa, respectively) compared to other tissues (from μ__ RF_ = 3.91 ± 0.16 kPa to μ__VI_ = 4.23 ± 0.25 kPa). Subcutaneous adipose tissue had the lowest value (μ__adipose tissue_ = 3.04 ± 0.12 kPa) of all the tissues tested.

**Conclusion:**

The different elasticities measured between the tissues may be due to variations in the muscles' physiological and architectural compositions. Thus, the present protocol could be applied to injured muscles to identify their behavior of elastic property. Previous studies on muscle pathology found that quantification of the shear modulus could be used as a clinical protocol to identify pathological muscles and to follow-up effects of treatments and therapies. These data could also be used for modelling purposes.

## Introduction

Muscles have mechanical properties that vary according to activity and the real-time estimation of a muscle's contractile properties is challenging. Elastography is a non-invasive *in vivo* technique that can provide reliable quantitative information on the mechanical properties of contracted or relaxed muscle, and could improve our understanding of their functional behavior.

Biopsies and palpations remain the conventional clinical tests used to diagnose and to monitor [1–3] muscle diseases, respectively. However, these techniques are invasive and subjective. Thus, elastographic methods, based on the propagation of shear waves in soft tissues, have been developed using magnetic-resonance imaging (MRI) [4–9] and ultrasound [7,10–13]. Elastography aims to provide reliable quantitative information on the mechanical properties (e.g., elasticity and viscosity) of skeletal muscles, which could indicate their functional behavior. Indeed, the myopathies that affect the composition and organization of injured muscles lead to different mechanical properties compared to those of healthy tissues.

Ultrasound elastography, such as a supersonic shear imaging (SSI) system, has been applied to the upper trapezius muscle during different shoulder positions to demonstrate the feasibility and sensitivity of changes to the muscle shear modulus measurements [12]. Similarly, SSI was also applied to the calf muscle with different ankle angles of dorsiflexion and plantarflexion [14] to measure changes in shear wave speed. Subsequently, SSI has been used to determine physiological muscle structures, such as the slack length of each head of the biceps brachii [15]. This microstructural analysis has shown that the electromechanical delay (i.e., the time-lag between muscle activation and force development) is related to muscle architecture [16]. Ultrasound can represent the displacement of muscle fibers and aponorosis structures compared to those found using MRI, which cannot provide information at the structural level of muscle fibers. However, deeper muscles are difficult to characterize with ultrasound elastography due to the depth limitation of the beam [10].

MR elastography (MRE) is based on MRI technology and can resolve both superficial and deep muscles in a single image [17]. Thus, the shear modulus of different muscles located within the same volume (such as the vastus medialis, vastus lateralis, or sartorius) can be simultaneously characterized [5,18]. MRE was first introduced by Muthupillai in 1995 [19], and there are now several MRE methods that can be applied to characterize the tissues of interest [20–22] using different mechanical excitations [5,23,24], data acquisition methods, and processing algorithms [25–28].

Ultrasound and MR elastography are complementary tools that can be performed on the same muscle sample. A comparison of the results has linked MRE shear wave displacement to muscle architecture (ultrasound acquisition) and has validated the physiological changes to muscle observed with MRE shear modulus mapping [7].

All muscles from the same group (i.e., thigh, calf, or arm) are not affected in the same ways by pathology or an injury. Therefore, there is a need to characterize the functional properties of isolated muscles. Previous MR and ultrasound elastography studies have performed these measurements within the upper (arm) [9,29] or lower (calf, thigh) [30–32] part of the body. Thus, the purpose of this study was to develop MRE protocols that could determine the shear modulus of the nine muscles in the thigh at rest. This present study is the first step in the development of a muscle atlas that could be used in clinical and modelling fields.

## Materials and Methods

### Ethics statement

**Fig 1:**
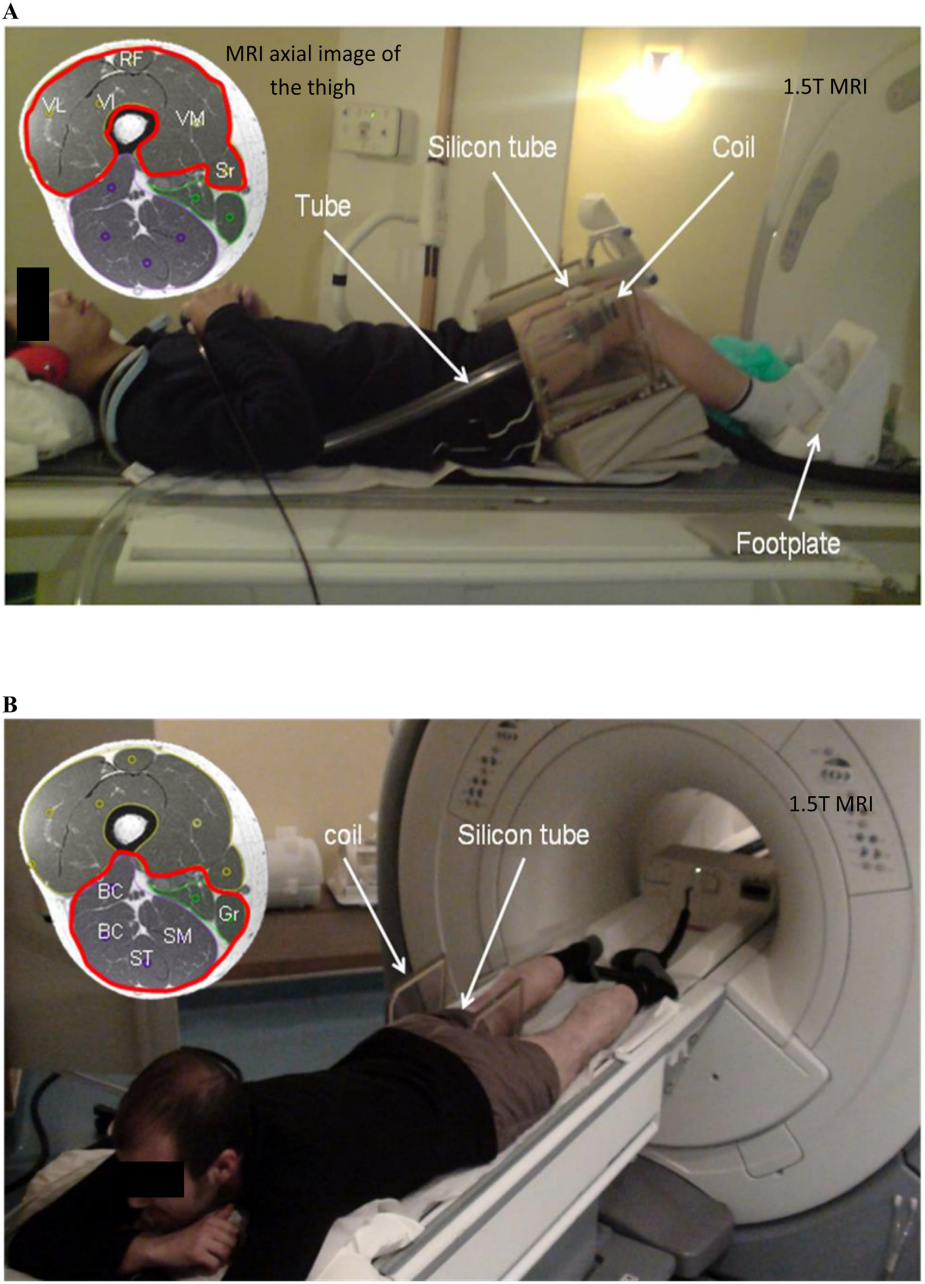
MRE setup placed inside a 1.5 T MRI machine. **A:** A participant laid supine on a custom-built ergometer to characterize the quadriceps (VL, RF, VI, and VM) and sartorius (Sr) muscles. **B:** Participant laid on his abdomen to analyze the ischio (ST, SM, BC) and gracilis (Gr) muscles. Waves were generated at 90 Hz through a pneumatic driver (silicon tube) attached around the thigh muscles, where a coil was placed. VL: vastus lateralis, RF: rectus femoris, VI: vastus intermedius, VM: vastus medialis, Sr: sartorius, ST: semitendinosus, SM: semimembranosus, BC: biceps, Gr: gracilis.

In this study, ethical approval to study human subjects was sought from the institutional review board of Amiens Hospital. All subjects had the experimental protocol explained and then gave their informed written consent prior to admission into the study. The individual in Fig 1, showing him during this experiment, gave his written informed consent, as outlined in PLOS consent form, to publish the figure.

### Participants

The thigh muscles from 29 healthy volunteers (7 women, 22 men, age range: 21–38 years, mean age = 26 ± 3.41 years, mean body mass index = 23.55 ± 3.31 kg.m^-2^(range: 18.93–33.12)) with no muscle abnormalities or histories of muscle disease were studied.

### Experimental configuration

MRE tests were conducted with a 1.5 T *Signa HDx* MRI machine (General Electric, Milwaukee, WI, USA). The quadriceps (rectus femorus: RF, vastus intermedius: VI, vastus medialis: VM, vastus lateralis: VL) and sartorius (Sr) muscles of subjects in the supine position were studied (Fig 1A). For the characterization of the group of ischio (semitendinous: ST, semimembranous: SM, biceps (long and short): BC) and gracilis (Gr) muscles, the subjects were placed in a prone position (Fig 1B). Although only the supine position could be used for all the investigated muscles, the prone position was used to later characterize the muscles in their active states using the following protocol.

Fig 1A shows a homemade ergometer that had been used in previous studies [5] and shows the right knee positioned at a 30° flexion with the right foot placed on a support and secured with Velcro straps. A custom-made Helmholtz surface coil was placed around the thigh, and a pneumatic passive driver (silicon tube), consisting of a remote active pressure driver connected to a hose (tube) was wrapped and clamped around the subject’s thigh.

For characterization of the quadriceps and sartorius muscles, the tube was placed on the lower part of the thigh because of the lesser thickness of adipose tissue in this area. Subsequently, the tube was moved to the middle part of the thigh to investigate the ischio and gracilis muscles; this enabled better placement of the driver above a larger volume of muscle. Periodic variations in air pressure were induced inside the tube at 90 Hz (frequency: f), resulting in the propagation of acoustic waves within the muscles. This frequency was chosen as optimal based on previous MRE experiments on thigh muscles with the present tube driver [5,33]. The MRE pulse sequence included a motion-encoding gradient that oscillated, in the Z-direction, at the same frequency as the driver (90 Hz), and was used to image the displacement of the shear waves.

### Acquisition of the anatomical and phase images

**Fig 2:**
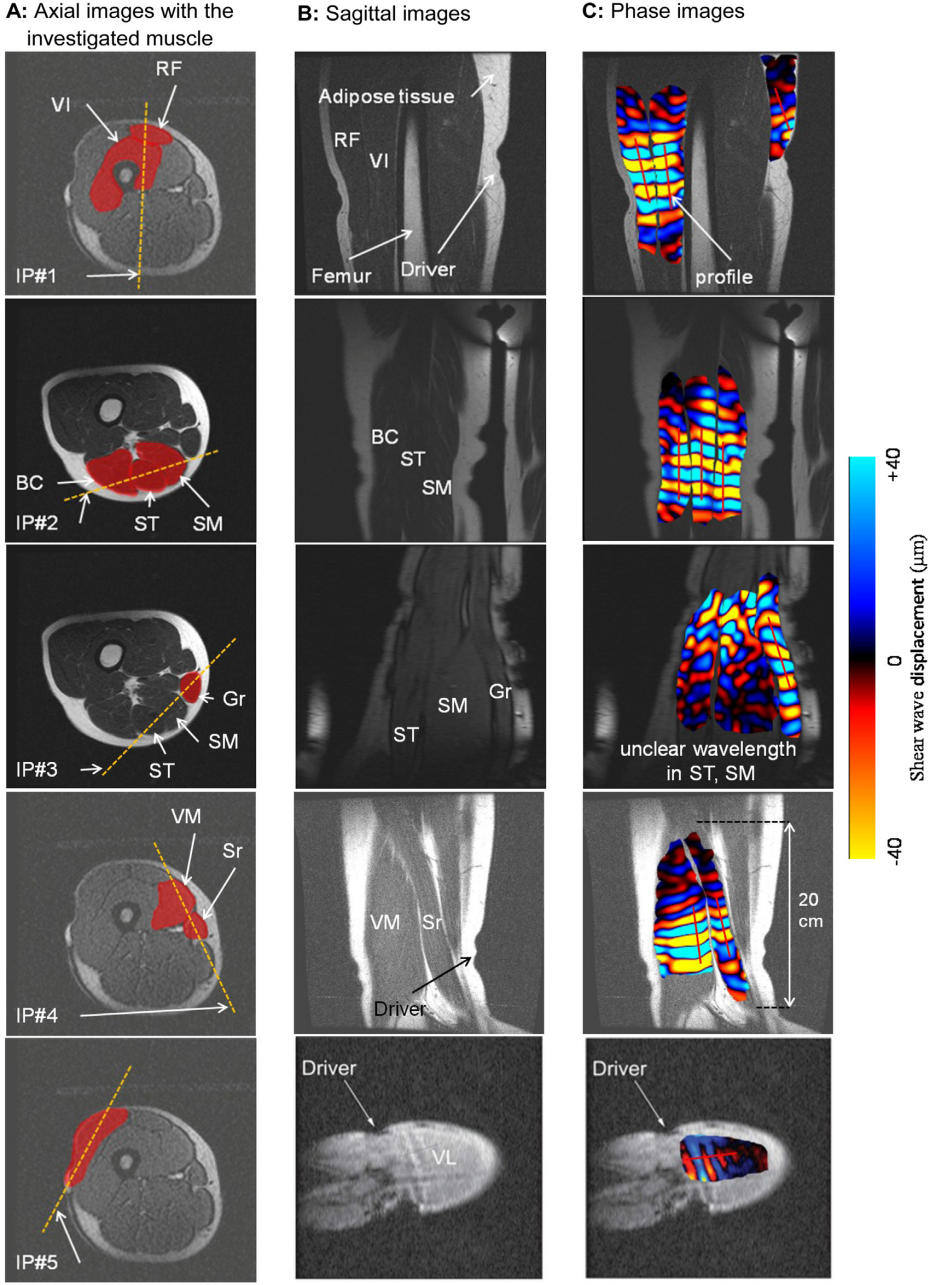
Illustration of the three MRE steps to obtain the phase image. **A** (step #1): The first column shows the baseline orientation of the imaging plane (IP) as represented by a dashed line, within the target muscle. **B** (step #2): Sagittal images were obtained from step #1 and represent the investigated muscles along the thigh. **C** (step #3): A MRE sequence was performed on the selected sagittal image leading to acquisition of the phase image, representing displacement of the shear waves within the muscle. VL: vastus lateralis, RF: rectus femoris, VI: vastus intermedius, VM: vastus medialis, Sr: sartorius, ST: semitendinosus, SM: semimembranosus, BC: biceps, Gr: gracilis.

A series of axial scout images of the thigh were acquired with a 2D gradient echo (GRE) MRE sequence. Five different orientations of imaging planes (IP) were manually positioned on the axial image of each muscle of interest. After several rotations of the imaging planes, the best ones used to obtain clear and consistent displacement of the waves for the different muscles were summarized at Fig 2A. Each orientation corresponded to a protocol to characterize specific muscles.

**Fig 3:**
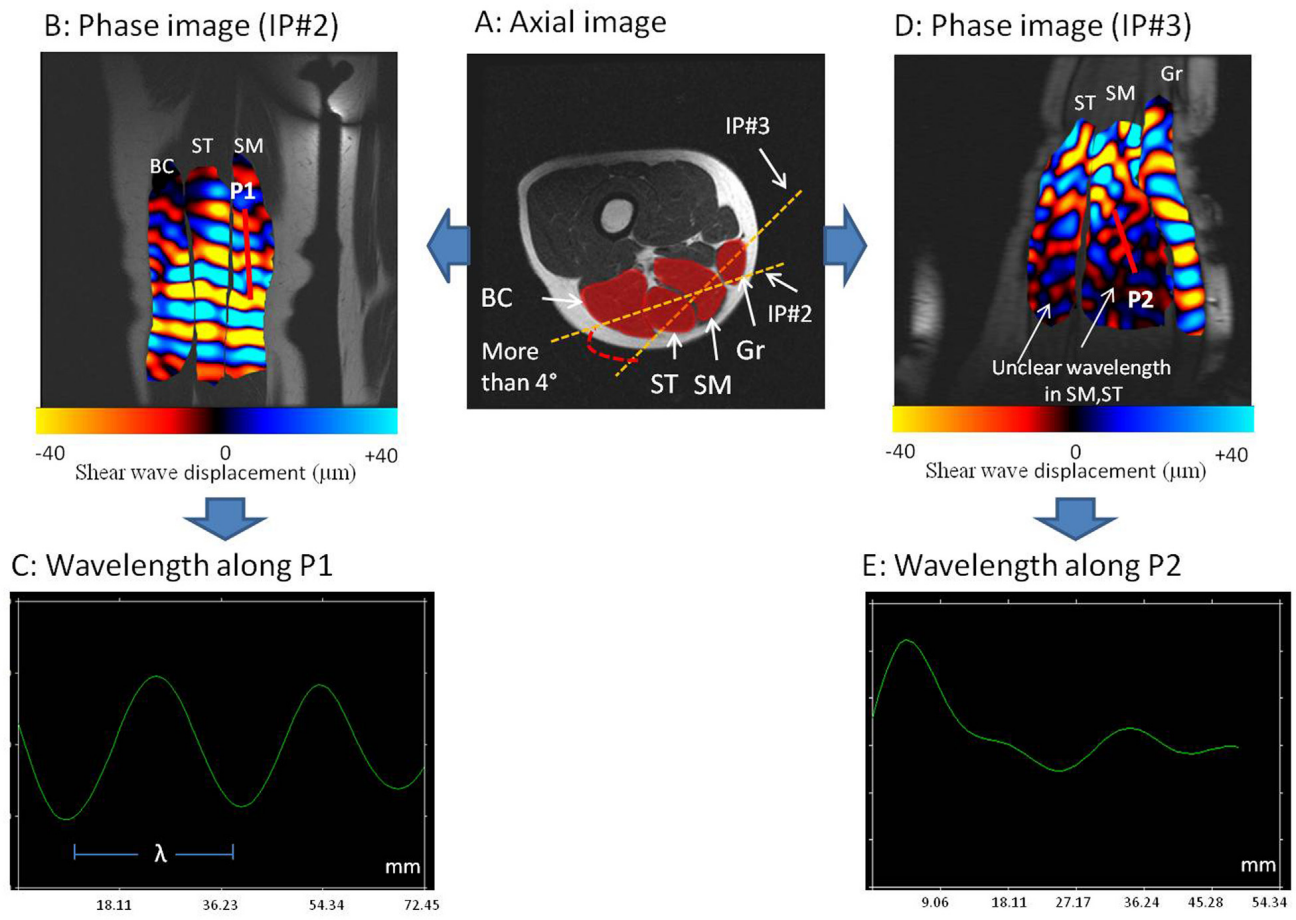
Visualization of clear and unclear wave propagation. A: Axial image with two different orientations of the imaging planes (IP#2, IP#3) through semimembranosus (SM) and semitendinosus (ST). Phase images showing clear (B) wave with measurable wavelength (λ) (C) and unclear (D) waves with non measurable wavelength (E). P1: Profile 1, P2: Profile 2.

The orientation of each imaging plane was slightly rotated (from the baseline orientation, Fig 2A) and it was found that a range of ±4 degrees, measured in the MRI console, was acceptable to visualize clear propagation of the wave. Fig 3 showed a clear wave displacement (Fig 3B) and unclear wave propagation when the imaging plane was rotated over ±4 degrees. Previous studies [7] have demonstrated that wave displacement was occurring primarily along the muscle fiber [34], which led to placement of the imaging plane parallel to the muscle fiber. Moreover, this orientation was specific to each muscle due to the orientation of the muscle fiber and to the localization of the muscle within the thigh.

Anatomical landmarks were represented through sagittal images (Fig 2B), which showed the shapes of the entire muscles along the thigh. This MRI anatomical muscle shape must be known in order to have clear wave propagation that can be represented by a measurable wavelength (Fig 3), and that can be tracked (Fig 2C). Moreover, the five sagittal images (Fig 2B) could be used as a reference for muscle shape to accurately place the imaging plane. The wave displacement phase image (Fig 2C) was recorded with a 256×256 acquisition matrix, two opposite polarities of the motion-encoding gradient with a 2.2 G/cm maximum gradient amplitude limit, a flip angle of 45°, a 24-cm field of view, and a slice thickness of 5 mm. Four offsets were recorded for the nine thigh muscles in a relaxed condition. For each imaging plane, the scan time was 40 s corresponding to a TR/TE of 56 ms/23 ms.

### Phase image processing and data analyses

The recorded phase images underwent post-processing by applying a mask, which removed the noise located in the background of the image. A directional filter, oriented along the direction of the wave propagation, combined with a Butterworth spatial filter, were applied to simultaneously remove interfering waves, longitudinal waves, and noise [35].

**Fig 4:**
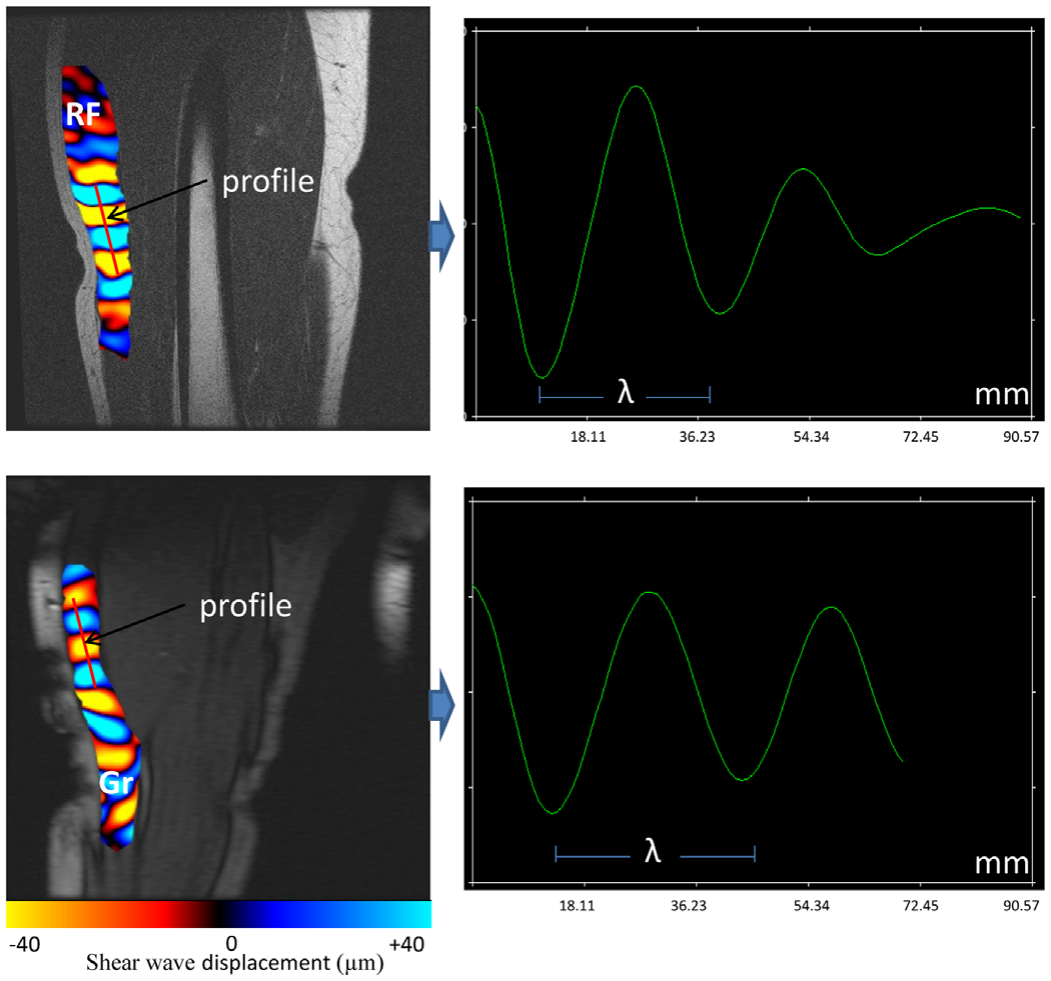
Behavior of the shear wave along the prescribed profile. The gracilis (Gr) muscle revealed a higher wavelength (λ) compared to the rectus femoris (RF) muscle.

The shear modulus (μ) of the different muscles and the adipose tissue surrounding the muscle were obtained from displacement of the shear wave. The wavelength (λ) that represented the distance between consecutive peaks (Fig 4) was quantified with a profile that was manually prescribed in the direction of shear wave propagation (since only the Z encoding direction was recorded) within the muscle and adipose tissues (Fig 2C).

Assuming that the muscle was linearly elastic, isotropic, homogeneous, and incompressible, the shear modulus (μ), which represented the local elasticity, was calculated using the following equation μ = ρ (f. λ)^2^ [19], where ρ is the muscle density and assumed to be close to that of water (i.e., 1000 kg/m^3^), f is the frequency and λ is the wavelength.

### Statistical analyses

A Friedman test and post-hoc t-test (Kolmogorov-Smirnov) were performed with Statgraphics 5.0 (Sigma Plus, Maryland, USA) software to compare the shear moduli between the muscles and the subcutaneous adipose tissues. The level of significance was set at *p* <0.05.

## Results

### Propagation of the shear waves within the nine muscles

Fig 2A shows the imaging planes used to obtain clear and consistent displacement of the waves for the different muscles. This result was obtained after several rotations of the imaging planes within the investigated muscles.

Imaging plane #1 (IP#1) was vertically placed through the rectus femoris (RF) and vastus intermedius (VI) muscles. IP#1 allowed visualization of wave displacement for both muscles during the same MRE test. Any reflective wave was obtained by the presence of the femur. By using imaging plane #2 (IP#2), all muscles from the ischio group (BC, ST, SM) were characterized in the same phase image (Fig 2C). Another imaging plane #3 (IP#3) was used to analyze the gracilis (Gr) muscle. The sagittal image revealed the gracilis, a long (20 cm) and thin muscle, near the SM and ST muscles. Moreover, unclear propagation was obtained through the SM and ST muscles with IP#3, demonstrating the importance of accurate placement of the imaging plane according to muscle architecture and localization within the thigh. Fig 3 summarized this last result.

The identification of the imaging plane for the vastus medialis (VM), sartorius (Sr) (IP#4), and vastus lateralis (VL) (IP#5) muscles has been previously determined [5]. These results are included here (Fig 2) to provide complete representation of the MRE protocol and characterization of these thigh muscles.

Characterization of the shear modulus of the adipose tissue was independent of the placement of the imaging plane. The physiological composition of the adipose tissue, being softer than the muscle, provided excellent propagation of the wave within this media around the thigh muscle. A profile was placed, within the subcutaneous adipose tissue layer, in the direction of the wave based on the phase image (Fig 2C).

### Comparison of the shear modulus between muscles at rest

**Fig 5:**
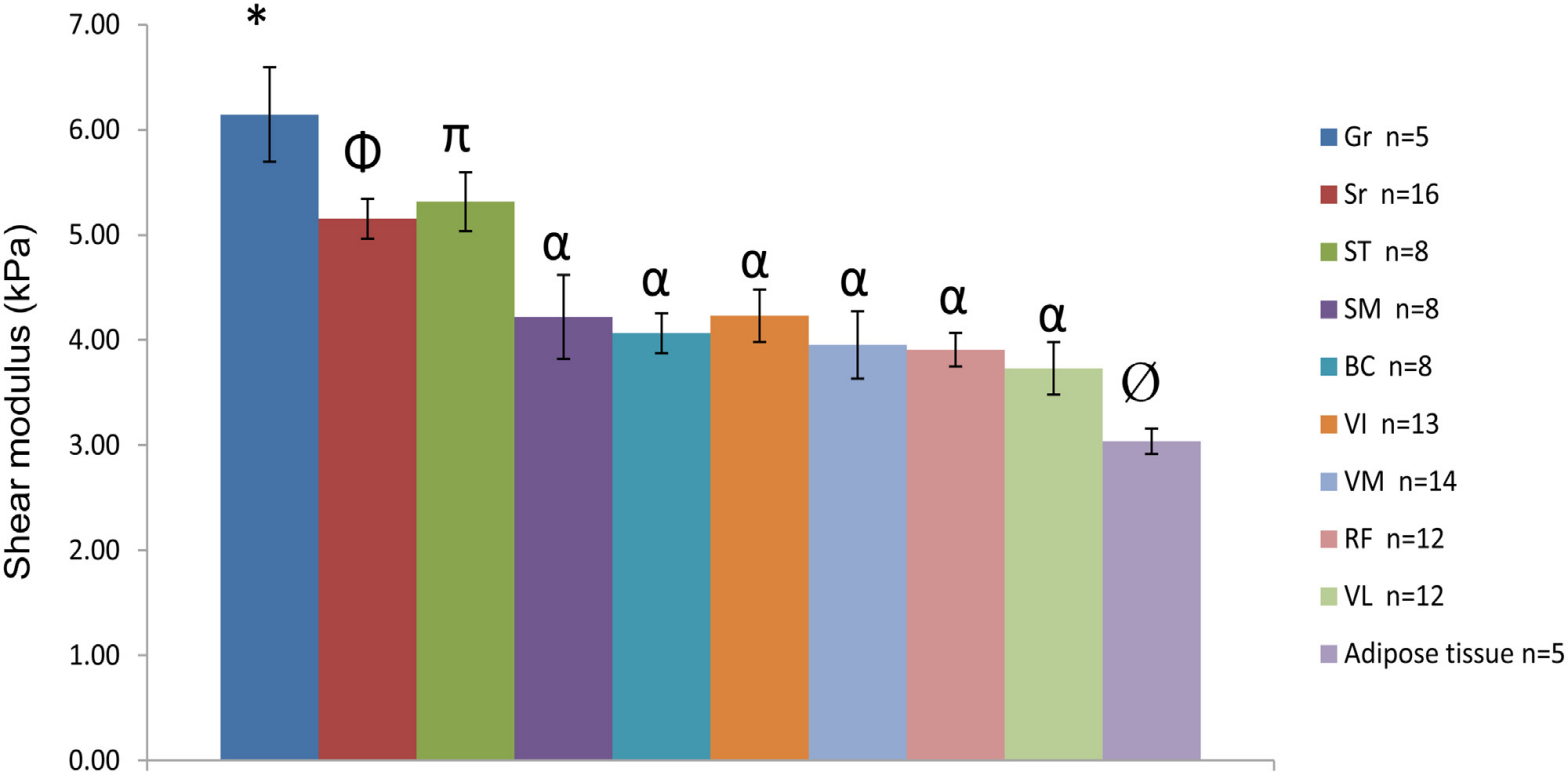
Shear modulus (μ) with SEM obtained for the different thigh muscles and adipose tissues. VL: vastus lateralis, RF: rectus femoris, VI: vastus intermedius, VM: vastus medialis, Sr: sartorius, ST: semitendinosus, SM: semimembranosus, BC: biceps, Gr: gracilis. *: significantly different to all tissues (*P* < 0.04). Φ: significantly different to all tissues except ST (*P* < 0.03). π: significantly different to all tissues except Sr (*P* < 0.03). α: significantly different to Gr, Sr, ST and adipose tissue (*P* < 0.04). Ø: significantly different to all muscles (*P* < 0.01).

Due to the high number of acquisitions and time limitations, it was not possible to apply all the imaging planes for each volunteer. Fig 5 shows the values of the shear moduli for the nine muscles and the subcutaneous adipose tissues. Different shear moduli values were found for the muscles in a relaxed state. The gracilis muscle had a significantly (*p* <0.04) higher shear modulus (μ__Gr_ = 6.15 ± 0.45 kPa) compared to the other tissues. Two muscles (Sr and ST) also showed significantly (*p* <0.03) higher shear moduli (μ__ Sr_ = 5.15 ± 0.19 kPa and μ__ ST_ = 5.32 ± 0.10 kPa) compared to the other five muscles (SM, BC, VI, VM, and RF) and the subcutaneous adipose tissue. These remaining tissues (SM, BC, VI, VM, and RF) had similar shear moduli (from μ__ RF_ = 3.91 ± 0.16 kPa to μ__VI_ = 4.23 ± 0.25 kPa). The shear modulus of the subcutaneous adipose tissue had the lowest (*p* <0.01) value (μ__adipose tissue_ = 3.04 ± 0.12 kPa) compared to all other muscles. The reproducibility of the shear moduli was assessed from successive MRE tests with a delay of 5 min between each test. The reproducibility of the measurements performed twice for all the muscles was evaluated through an intraclass correlation coefficient (ICC) [36]. The result showed an average ICC of ≈ 0.8 (range from 0.72 to 0.87) for the nine muscles and the subcutaneous adipose tissue, attesting to the good reproducibility of the shear moduli.

## Discussion

The development of non-invasive protocols to assess functional tissue properties has provided useful information. This study shows the feasibility of developing MRE muscle protocols to quantify the shear modulus of all nine healthy thigh muscles. It could be used as a method to identify problems within injured or diseased muscles and to follow the effects of treatments and therapies. These data, obtained from healthy resting muscles, could be used as a baseline model by clinicians. Moreover, human *in vivo* data for modeling the mechanical properties of the muscle are lacking. Thus, our obtained values will be of interest for modeling purposes in implementing the true *in vivo* characteristics of lower limb muscles.

In 2006, the first MRE protocol was published to characterize the vasti (VL, VM) and sartorius (Sr) muscles [5]. This previous protocol used the same tube driver and similar imaging planes as the present study. Similar ranges of the shear modulus for the vasti (VL, VM) (from 3 kPa to 4 kPa) and Sr (from 5.5 kPa and 7.5 kPa) were found in both studies. Other studies have established MRE muscle protocols with different drivers and MR sequences to also determine the mechanical properties (i.e., elasticity and viscosity) of individual muscles as a function of age, muscle condition [8,37], and diseases [33], such as myopathy [4,38]. The originality of the present study was to characterize all the thigh muscles using the same MRE set up. In cases of muscle pathologies or injuries, it appears that different muscles have different physiological and functional changes. Thus, it was important to develop other muscle protocols that allowed us to assess the complete behavior of a whole group of muscles. These shear moduli data will improve our understanding of the changes in muscle elasticities at rest and during contraction, and will enable us to adapt treatments as a function of muscle damage.

One of the challenges of developing a successful MRE technique is to characterize deep muscles. This method is based on the interpretation of wave propagations along the muscle fibers. Although this architecture is not available in MRE, ultrasound can reveal the orientation of muscle fibers [7] and their anisotropic behavior [39], through rotation of the probe. Nevertheless, ultrasound is limited by the depth penetration of the beam [10]. The present study has demonstrated the ability and potential of MRE to quantify the shear modulus of deep muscles in the thigh, at rest. In addition, MRI provides a larger field of view compared to ultrasound and can reveal a complete view of the muscle (e.g., gracilis) from the distal to the proximal sides of the thigh. This representation is of importance and can identify the affected muscle area and localize future treatments such as pharmaco-gene therapeutic trials for Duchenne muscular dystrophy [38].

The present study has demonstrated different shear moduli values between the muscles. The gracilis, sartorius, and semitendinosus muscles showed higher shear modulus compared with the other thigh muscles. Similar trends were also found for the sartorius and semitendinosus muscles. These differences in shear moduli could have been caused by the position (supine vs prone), the subject's knee angle, and the placement of the scaning planes. However, the muscles (Gr, Sr) were tested in both positions and revealed similar mechanical properties, demonstrating that position had no influence on these two muscles. It is thus assumed that the physiological and architectural compositions of the muscles have impacts on wave displacement. Moreover, the lower shear modulus for the adipose tissue was may be due to its physiological composition which differed to that of muscle tissue [7]. To better analyze the physiological and structural composition of the different muscles, diffusion [40–43] imaging technique could be performed. Moreover, it would be of interest to couple the MR elastographic sequence with diffusion imaging to characterized the anisotropic [32,44,45] properties when there is muscle disease [34]. In this way, the anisotropic behavior of muscle could be characterized and so avoid the previous isotropic assumption. To appreciate the real mechanical behavior of muscle, the viscoelastic properties could be also measured using rheological models.

The setup of the present MRE muscle protocols is mainly related to the orientation of the imaging planes, which are important in achieving good wave displacement. The good reproducibility we found showed the reliability of our study. This work is the first step towards the development of a muscle atlas that could be improved by including other parameters, such as viscosity [18,31,45], anisotropy [32], fiber type, muscle volume, and percentages of water and adipose tissues.

The present MRE protocols were developed for muscles at rest condition and will be applied for active muscles condition. According to previous studies, it is expected that the shear moduli will increased as a function of the level of contraction [8].

Future perspectives will be to determine the active muscle shear modulus for all thigh muscles. The present MRE methods to measure muscles can be used as a non-invasive diagnostic tool to evaluate alterations to tissues or the progression of disease and the effect of treatments, such as those in the ongoing therapeutic trials on Duchenne muscular dystrophy [38].

## Supporting Information

S1 Table Shear modulus (μ) with SEM for the nine thigh muscles and the adipose tissues. (XLS)
