# Correction: Quantifying the Elastic Property of Nine Thigh Muscles Using Magnetic Resonance Elastography

**DOI:** 10.1371/journal.pone.0142958

**Published:** 2015-11-10

**Authors:** The PLOS ONE Staff

## Notice of Republication

S1 Table was published in error. The error was corrected in the HTML and PDF versions of this article on October 29, 2015, and the corrected table is available in the supporting information of this article. The publisher apologizes for the error.

## Supporting Information

S1 File Corrected S1 Table. (XLS)
